# Combination of Sulindac and Dichloroacetate Kills Cancer Cells via Oxidative Damage

**DOI:** 10.1371/journal.pone.0039949

**Published:** 2012-07-17

**Authors:** Kasirajan Ayyanathan, Shailaja Kesaraju, Ken Dawson-Scully, Herbert Weissbach

**Affiliations:** 1 Center for Molecular Biology and Biotechnology, Charles E. Schmidt College of Science, Florida Atlantic University, Jupiter, Florida, United States of America; 2 Department of Biological Sciences, Charles E. Schmidt College of Science, Florida Atlantic University, Boca Raton, Florida, United States of America; Bauer Research Foundation, United States of America

## Abstract

Sulindac is an FDA-approved non-steroidal anti-inflammatory drug with documented anticancer activities. Our recent studies showed that sulindac selectively enhanced the killing of cancer cells exposed to oxidizing agents via production of reactive oxygen species (ROS) resulting in mitochondrial dysfunction. This effect of sulindac and oxidative stress on cancer cells could be related to the defect in respiration in cancer cells, first described by Warburg 50 years ago, known as the Warburg effect. We postulated that sulindac might enhance the selective killing of cancer cells when combined with any compound that alters mitochondrial respiration. To test this hypothesis we have used dichloroacetate (DCA), which is known to shift pyruvate metabolism away from lactic acid formation to respiration. One might expect that DCA, since it stimulates aerobic metabolism, could stress mitochondrial respiration in cancer cells, which would result in enhanced killing in the presence of sulindac. In this study, we have shown that the combination of sulindac and DCA enhances the selective killing of A549 and SCC25 cancer cells under the conditions used. As predicted, the mechanism of killing involves ROS production, mitochondrial dysfunction, JNK signaling and death by apoptosis. Our results suggest that the sulindac-DCA drug combination may provide an effective cancer therapy.

## Introduction

Sulindac is an FDA-approved non-steroidal anti-inflammatory drug (NSAID), which has also been shown to have anti-cancer activity [1]–[6]. Recent studies from our laboratory have demonstrated that RKO, A549 and SCC25 cancer cell lines exhibited sensitivity towards a combination of sulindac and an oxidizing agent, such as TBHP or H_2_O_2_ [7]. The data indicated that the sulindac effect was not related to its NSAID activity but that sulindac made cancer cells more sensitive to oxidative stress resulting in mitochondrial dysfunction and loss of viability. In contrast, normal cells did not show enhanced killing under similar conditions [7]. In the past 10 years there have been scattered reports of enhanced cancer killing using sulindac in combination with a variety of compounds including arsenic trioxide, bortezomib, difluoromethylornithine (DFMO) and suberoylanilide hydroxamic acid (SAHA) [8]–[14]. Although these compounds have different sites of action, a common mechanism for the sulindac/drug combination enhanced killing might involve oxidative damage, as was clearly demonstrated in our previous studies using sulindac and an oxidizing agent [7], [15]. In fact, ROS have been implicated in the studies using sulindac in combination with arsenic trioxide, bortezomib and SAHA [10], [12], [14].

Our previous results suggested that the enhanced killing of cancer cells by the combination of sulindac and an oxidizing agent might be due to a defect in respiration in cancer cells, as first described by Warburg more than 50 years ago [16], who noted that cancer cells favor glycolysis, not respiration, to obtain energy, unlike normal cells. Some cancer cells obtained as much as 50% of their energy from glycolysis, whereas glycolysis in normal cells account for less than 5% of the energy requirement [16]. To obtain further evidence for the possible roles of altered respiration and ROS in the killing of cancer cells by sulindac and oxidative stress, we initiated studies with sodium dichloroacetic acid (DCA). DCA is an ideal candidate as it is known to inhibit a kinase that down regulates the activity of pyruvate dehydrogenase, resulting in a shift of pyruvate metabolism away from lactic acid formation, towards respiration [17], [18]. DCA has been used clinically to treat patients with lactic acidosis [19], and based on its biochemical properties DCA has also been tested as an anticancer agent. Bonnet et al. 2007 have shown that DCA reverses the Warburg effect in cancer cells by redirecting cancer cell metabolism from glycolysis to oxidative phosphorylation. In these previous studies it was shown that DCA increases levels of ROS from complex I. This in turn triggers “ remodeling” of mitochondrial metabolism (reduces ΔΨm, opens mitochondrial transition pore) in cancer cells pushing them towards apoptosis. Furthermore, several recent studies have verified that DCA can increase ROS levels in cancer cells and depolarize the mitochondria membrane in lung, endometrial, and glioblastoma cell lines resulting in apoptosis both *in vitro* and *in vivo* [18], [20]–[22]. Of interest was the observation that under the conditions used DCA did not appear to significantly affect mitochondrial metabolism or viability in normal cells [18], [23].

Based on our previous observations on the cancer killing effect of sulindac and an oxidizing agent that affected mitochondrial metabolism [7], we postulated that the combination of sulindac and DCA could synergistically enhance cancer killing and have important therapeutic value. In the present study we have examined the effect of using sulindac in combination with DCA on the viability of A549 and SCC25 cancer cell lines. We have also studied the role of mitochondrial function and apoptosis in the cancer killing observed with this drug combination.

## Materials and Methods

### Materials

Sulindac, N-acetylcysteine and Tiron were purchased from Sigma (St.Louis, MO). DCA sodium salt was obtained from Acros Organics (Geel, Belgium). H2DCFDA and JC-1 were purchased from Molecular Probes (Eugene, OR). MTS assay reagent and Deadend Tunel Kit were obtained from Promega (Madison, WI). Cytosol/mitochondria fractionation kit and the CBA077 InnoCyte™ Flow Cytometric Cytochrome *c* Release kit were from Calbiochem, Gibbstown, NJ. All cell culture media, fetal bovine serum, and other supplements such as penicillin/streptomycin, glutamine, etc. were purchased from American Type Culture Collection (ATCC; Rockville, MD).

**Figure 1:**
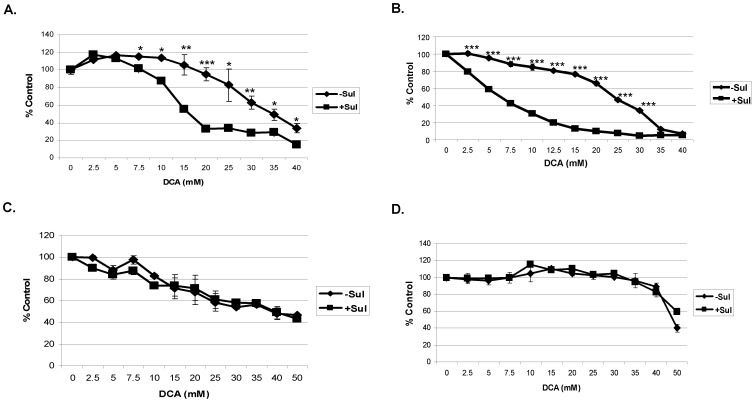
Sulindac in combination with DCA selectively kills cancer cells. The A549 and SCC25 cancer cells, normal lung cells, and human epidermal keratinocytes were treated with the indicated concentrations of DCA in the presence or absence of sulindac (Sul) for 48 hours. The cell viability was monitored by MTS assay as mentioned in the Methods. The cell viability is expressed as % of control (cells not treated with sulindac). Error bars are standard error of the mean (SEM) expressed as % of the mean value of quadruplicates from a representative experiment. Cell viabilities are illustrated for A549 cancer cells (A), SCC25 cancer cells (B), normal lung cells (C) and normal human epidermal keratinocytes (D). ♦, -Sul; ▪, + Sul. * p<0.05, ** p<0.005, ***p<0.0005.

### Cell Culture

A non small cell lung carcinoma cell line (NSCLC), A549, the normal human lung cell line, MRC-5, and a tongue-derived squamous cell carcinoma line, SCC25 were purchased from ATCC (Rockville, MD) and maintained in F12-K medium supplemented with 10% fetal bovine serum, 2 mM glutamine, 100 IU/ml penicillin, and 100 µg/ml streptomycin in a humidified, 5% CO_2_ incubator at 37°C. Normal human epidermal keratinocytes were obtained from Promocell GmbH (Heidelberg, Germany) and maintained in the recommended culture medium. Early passage, non-immortalized normal cells were used for the experiments.

**Figure 2:**
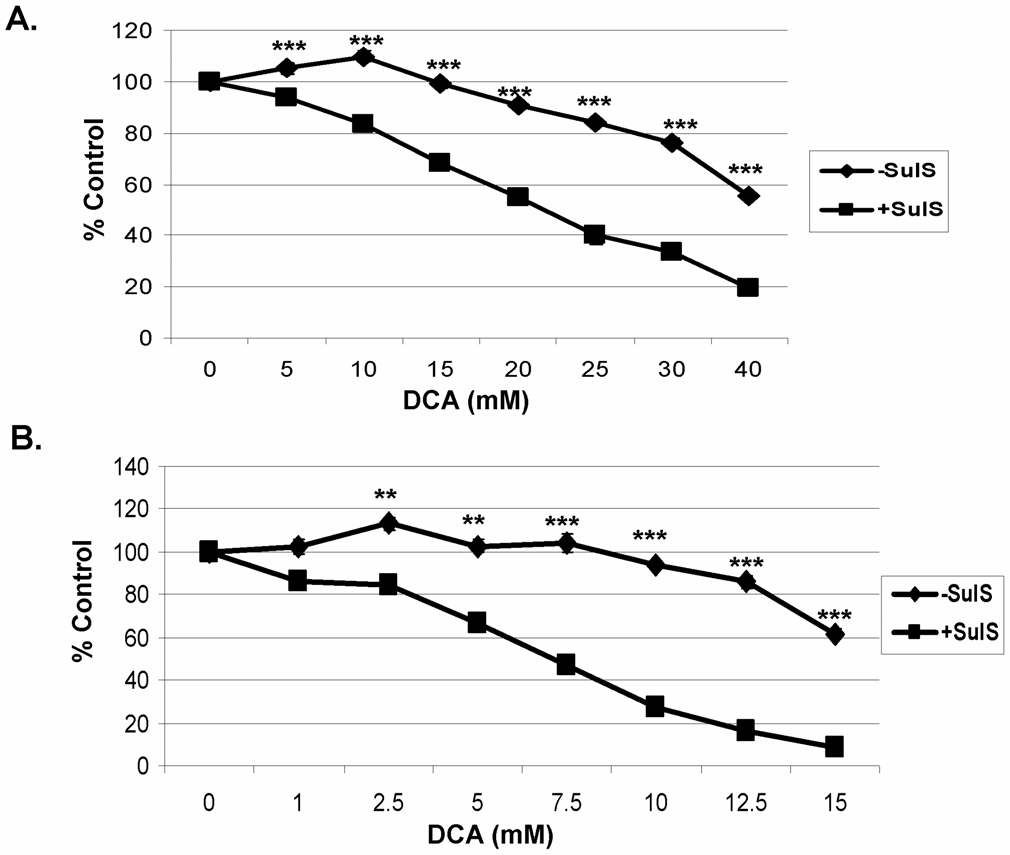
Sulindac sulfone in combination with DCA also kills cancer cells. The A549 and SCC25 cancer cells were treated with the indicated concentrations of DCA in the presence or absence of sulindac sulfone (SulS) for 48 hours. SulS was used at a final concentration of 250 µM for A549 cancer cells and 75 µM for SCC25 cancer cells. The cell viability was monitored by MTS assay as described in Methods. The cell viability is expressed as % of control (cells not treated with sulindac sulfone). Error bars are standard error of the mean (SEM) expressed as % of the mean value of triplicates from a representative experiment. Cell viabilities are illustrated for A549 cancer cells (A) and SCC25 cancer cells (B). ♦, -SulS; ▪, + SulS. * p<0.05, ** p<0.005, ***p<0.0005.

### Cell Viability Assay

The A549 cancer and lung normal cells were plated at 3×10^3^ cells per well while SCC25 cancer cells and normal keratinocytes were plated at 7.5×10^3^ cells per well in a 96-well plate. The cells were grown for 18–20 hours, the medium discarded in aseptic conditions and replaced with fresh culture medium containing the indicated drug combinations. Where indicated 500 µM sulindac was used with the A549 cancer and lung normal cells and 100 µM sulindac was used with SCC25 cancer and normal keratinocyte cells. The plates were incubated for 48 hours at 37°C in a 5% CO_2_ incubator. The culture medium was discarded and the cells were thoroughly rinsed in 1× PBS. Cell viability was determined by using the CellTiter 96 Aqueous One Cell Proliferation Assay (Promega) according to the manufacturer’s instructions. The assay utilizes a tetrazolium compound that is converted into a water-soluble formazan by the action of cellular dehydrogenases present in the metabolically active cells [24]. The formazan was quantified by measuring the absorbance at 490 nm using a colorimetric microtiter plate reader (SpectraMax Plus; Molecular Devices). Background absorbance was subtracted from each sample.

### Intracellular Measurement of ROS

The A549 and SCC25 cancer cell lines were plated as above. Following the 48 hr drug treatment, the cells were incubated with 50 µM of dichlorodihydrofluorescein diacetate (H_2_DCFDA, Molecular Probes) in indicator free medium for 30 min at 37°C. Cells were rinsed with PBS and ROS levels were visualized by fluorescence microscopy. The images were captured using the Qcapture software and processed in Adobe photoshop. Image analysis was done using the slidebook software. Data obtained from a representative experiment were used for the quantification of DCF-positive cells as measured by the green fluorescence due to oxidized DCF.

**Figure 3:**
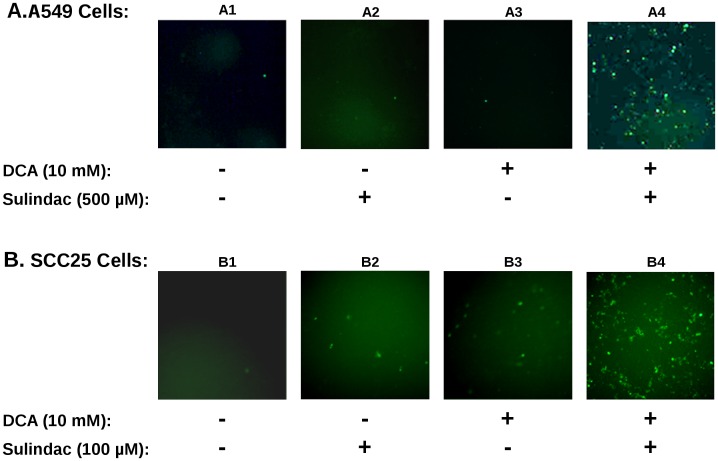
The combination of sulindac and DCA increases intracellular ROS levels in A549 and SCC25 cancer cells. Top panels (A) illustrate the results for A549 cancer cells while the bottom panels (B) depict the results for SCC25 cancer cells. The cells were treated with the indicated concentrations of drugs and processed for fluorescent microscopy as described in the Methods. The extent of intracellular ROS levels are illustrated as intensity of green fluorescence observed in cells treated with no drugs (sub-panels A1 and B1), sulindac alone (sub-panels A2 and B2), DCA alone (sub-panels A3 and B3), and sulindac and DCA combination treatment (sub-panels A4 and B4). Several independent fields were photomicrographed and representative fields for each condition are shown.

### JC-1 Staining to Monitor Mitochondrial Membrane Potential

Mitochondrial membrane potential was determined using the JC-1 dye (Molecular Probes). The A549 and SCC25 cancer cell lines were plated as above. Following the 48 hr drug treatment, the cells were incubated with 5 ng/ml of JC-1 dye in indicator free medium for 30 min at 37°C. Cells were rinsed with PBS and visualized by fluorescence microscopy. Normal mitochondria actively take up JC-1 dye in a potential-dependent manner and form J-aggregates, which gives a red fluorescence. Disruption and subsequent loss of mitochondrial membrane potential leads to increased green fluorescence in the cytosol due to monomeric JC-1, which is determined by following the appearance of green fluorescence using an FITC filter (Zeiss inverted microscope-Axiovert 40 CFL). Image capturing, processing, and analysis were performed as above. Data obtained from a representative experiment were used for the quantification of JC-1-green positive cells.

**Figure 4:**
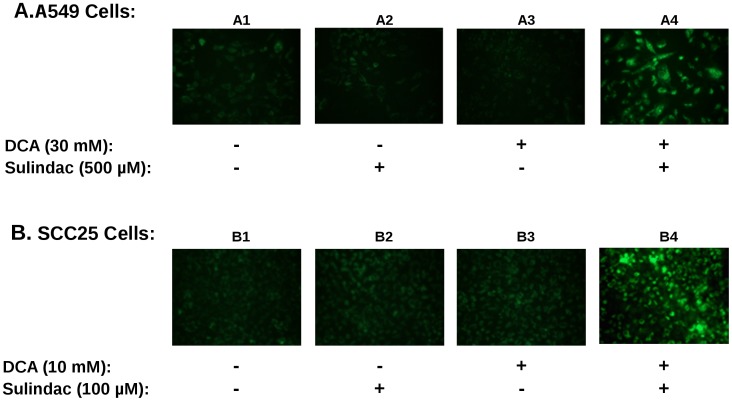
The combination of sulindac and DCA causes disruption of the mitochondrial membrane potential in cancer cells. Top panels (A) illustrate the results for A549 cancer cells while the bottom panels (B) depict the results for SCC25 cancer cells. Mitochondrial membrane potential loss was detected by a change in JC-1 distribution resulting in an increase in green fluorescence (see Methods). The experimental conditions for JC-1 staining and fluorescent microscopy are explained in detail under Methods and the drug treatment regimens are depicted below the panels. Untreated cells (sub-panels A1 and B1), cells treated with sulindac (sub-panels A2 and B2), cells treated with DCA (sub-panels A3 and B3), and cells treated with sulindac and DCA (sub-panels A4 and B4). Several independent fields were photomicrographed and representative fields for each condition are shown.

### Effect of ROS Scavengers on Cell Viability in the Presence of Sulindac and DCA

The A549 and SCC25 cancer cell lines were plated as described above. To scavenge ROS, either 2 mM N-acetylcysteine (NAC) or 2 mM Tiron (4,5-dihydroxy-1,3-benzenedisulfonic acid disodium salt) was added along with sulindac and DCA for 48 h at 37°C. Cell viability was monitored by the MTS assay and statistical analysis performed as mentioned above.

### TUNEL Staining to Monitor Cells Undergoing Apoptosis

TUNEL assay was performed in 96 well plates using the DeadEnd colorimetric tunel assay kit (Promega) following the manufacturer’s protocol. The A549 and SCC25 cancer cell lines were plated as above and treated for 48 hr with no drug, sulindac, DCA, or drug combination. Subsequent to drug treatment, the cells were fixed with formalin and permeabilized with 0.2% Triton X-100 in PBS. Cells were incubated with recombinant terminal deoxynucleotidyl transferase (TdT) and biotinylated nucleotides. Endogenous peroxidases were blocked with 0.3% H_2_O_2_ prior to the incubation with horseradish peroxidase-streptavidin (HRP-streptavidin) that binds to the biotinylated nucleotides incorporated into the nicked ends present in cells undergoing apoptosis. HRP-streptavidin labeled cells were detected by hydrogen peroxide and diaminobenzidine (DAB). Cells that show dark brown nuclear staining are indicative of apoptosis.

**Figure 5:**
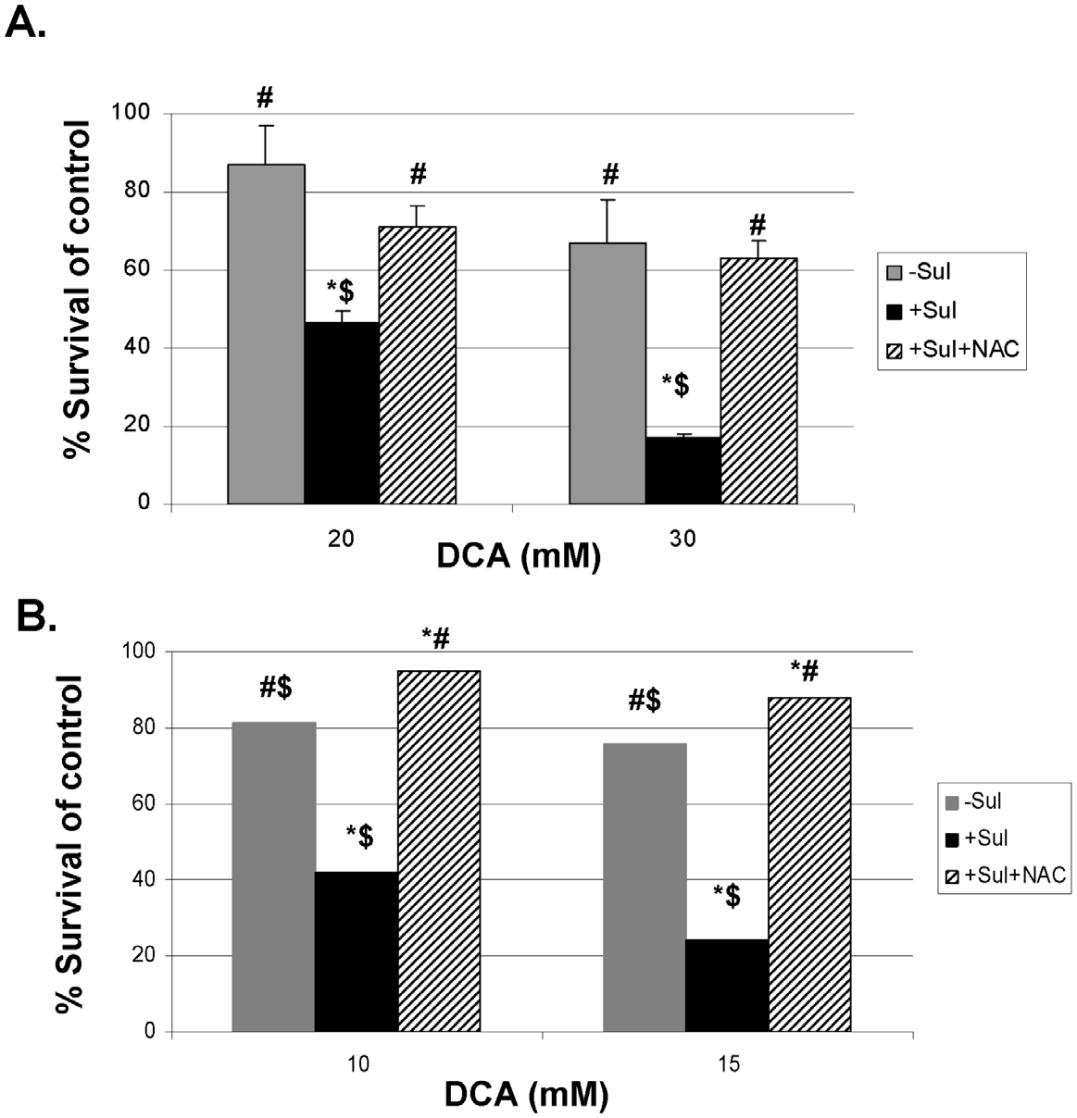
The ROS scavenger NAC reverses the killing of cancer cells by the combination of sulindac and DCA. The A549 and SCC25 cancer cells were treated with the indicated concentrations of DCA in the absence (grey bar) or presence of sulindac (black bar) or presence of sulindac and N-acetylcysteine (striped bar) for 48 hours. The cell viability was monitored by MTS assay as mentioned in the Methods. The cell viability is expressed as % of control (cells not treated with sulindac). Error bars are standard error of the mean (SEM) expressed as % of the mean value of quadruplicates from a representative experiment. Inhibition of cancer cell growth occurred in a dose dependent manner during combination treatment of DCA and sulindac (black bars) in both A549 cancer (A) and SCC25 cancer cells (B). However, this enhanced killing was prevented when N-acetylcysteine was present with the drug combination treatment (striped bars in A and B).

### Western Blot Analysis

Cells were grown to 70% confluency, treated with specified drugs for the indicated durations, and cytosolic fractions were isolated using the cytosol/mitochondria fractionation kit (Calbiochem, Gibbstown, NJ) following the manufacturer’s protocol. Briefly, cells were harvested at different time points and were then centrifuged at 600×g for 5 min at 4°C. The pelleted cells were suspended into the supplied buffer and incubated for 10 min on ice. The cells were then homogenized using a glass douncer and the homogenate centrifuged at 700×g for 10 min at 4°C to sediment nuclei and cell debris. The supernatant was spun at 10, 000×g for 30 min at 4°C to obtain the mitochondrial pellet and the supernatant was considered as the cytosolic fraction. Protein concentration was determined using a standard Bradford assay.

Sixty micrograms of total protein was loaded and separated on a 4–12% NuPage Bis-Tris gels (Invitrogen, Eugene, OR) and transferred onto a PVDF membrane that was probed by the primary antibodies. The primary antibodies, JNK, pJNK, cytochrome *c*, and PARP (Cell Signaling Technology, Danvers, MA), were used at 1∶1000 dilution. β-actin, (Santa Cruz Biotechnologies, Santa Cruz, California), was used at 1∶4000 dilution. Horseradish peroxidase conjugated secondary antibodies were used and bands were visualized using an enhanced chemiluminescence method (GE Healthcare, Piscataway, NJ).

**Figure 6:**
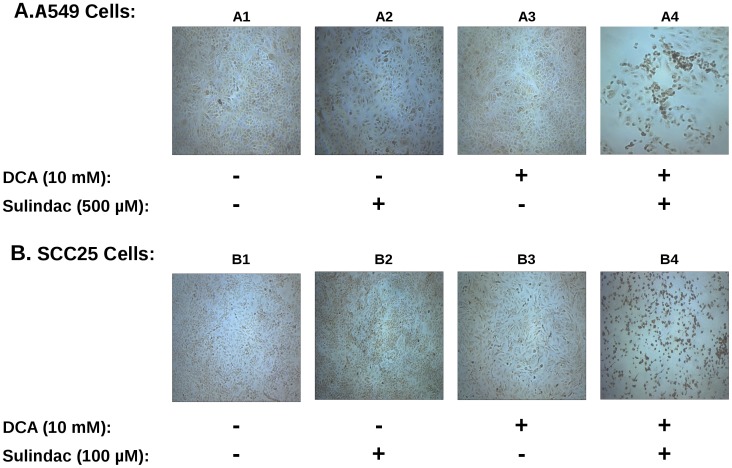
Sulindac in combination with DCA induce apoptosis in cancer cells. Top panels (A) illustrate the results for A549 cancer cells while the bottom panels (B) depict the results for SCC25 cancer cells. The extent of cells undergoing apoptosis was monitored by TUNEL staining of cells treated with no drugs (sub-panels A1 and B1), sulindac alone (sub-panels A2 and B2), DCA alone (sub-panels A3 and B3), and sulindac and DCA (sub-panels A4 and B4). The cells were treated with the indicated drugs as mentioned in the panels, subjected to TUNEL staining, and processed for fluorescent microscopy as described in the Methods. Several independent fields were photomicrographed and representative fields for each condition are shown. Brown-stained cells are indicative of cells undergoing apoptosis.

### Ligation-mediated PCR based DNA Laddering Assay to Monitor Extent of Cells Undergoing Apoptosis

To confirm the extent of apoptosis, ligation-mediated PCR based nucleosomal DNA laddering assay was performed as described [25]. The A549 and SCC25 cancer cell lines were plated at 5×10^4^ and 1×10^5^ cells per well in 35 mm dishes. The A549 cancer cells were treated for 48 hours with a) no drug, b) 500 µM sulindac, c) 20 mM DCA, and d) 500 µM sulindac plus 20 mM DCA. Similarly, SCC25 cancer cells were treated with the abovementioned four different drug combinations except that sulindac and DCA were used at 100 µm and 10 mM concentrations, respectively. After treatment, total cellular DNA were extracted, ligated to the adaptor constructed from 27-mer 5′-GACGTCGACGTCGTACACGTGTCGACT-3′ and 12-mer 5′- AGTCGACACGTGTAC-3′. Subsequent to ligation, the DNA was heated to release the 12-mer, filled with Taq polymerase, subjected to semi-quantitative PCR, and analyzed on a 1.2% agarose gel along with size markers.

### *In situ* Localization of Cytochrome *c* by Immunofluorescence

Intracellular location of cytochrome *c* was monitored by immunofluorescence by using the CBA077 InnoCyte™ Flow Cytometric Cytochrome *c* Release Kit according to the manufacturer’s instructions. Briefly, the SCC25 cells were plated at 3.5×10^5^ cells per 35 mm glass bottom dish and treated with the indicated drugs for 15 h. Cells were rinsed in 5 ml of 1× PBS and permeabilized on ice for 10 min in 300 µl of supplied buffer. The cells were fixed at RT for 20 min in 500 µl of 4% paraformaldehyde. Subsequent to washing and blocking, the cells were incubated with 250 µl of anti-cytochrome c antibody (1∶500 dilution) for 1 hr at RT. After washing, the cells were incubated with 300 µl of FITC-IgG (1∶300 dilution) for 1 hr at RT. Finally, the cells were stained with 300 µl of DAPI (1 mg/ml) for 10 min at RT. Cells were visualized using an Olympus inverted fluorescent microscope. Images were captured and processed as mentioned above. Several fields were analyzed and representative micrographs showing the localization patterns of cytochrome *c* under each treatment condition were obtained. Quantitative values are presented in the text.

**Figure 7:**
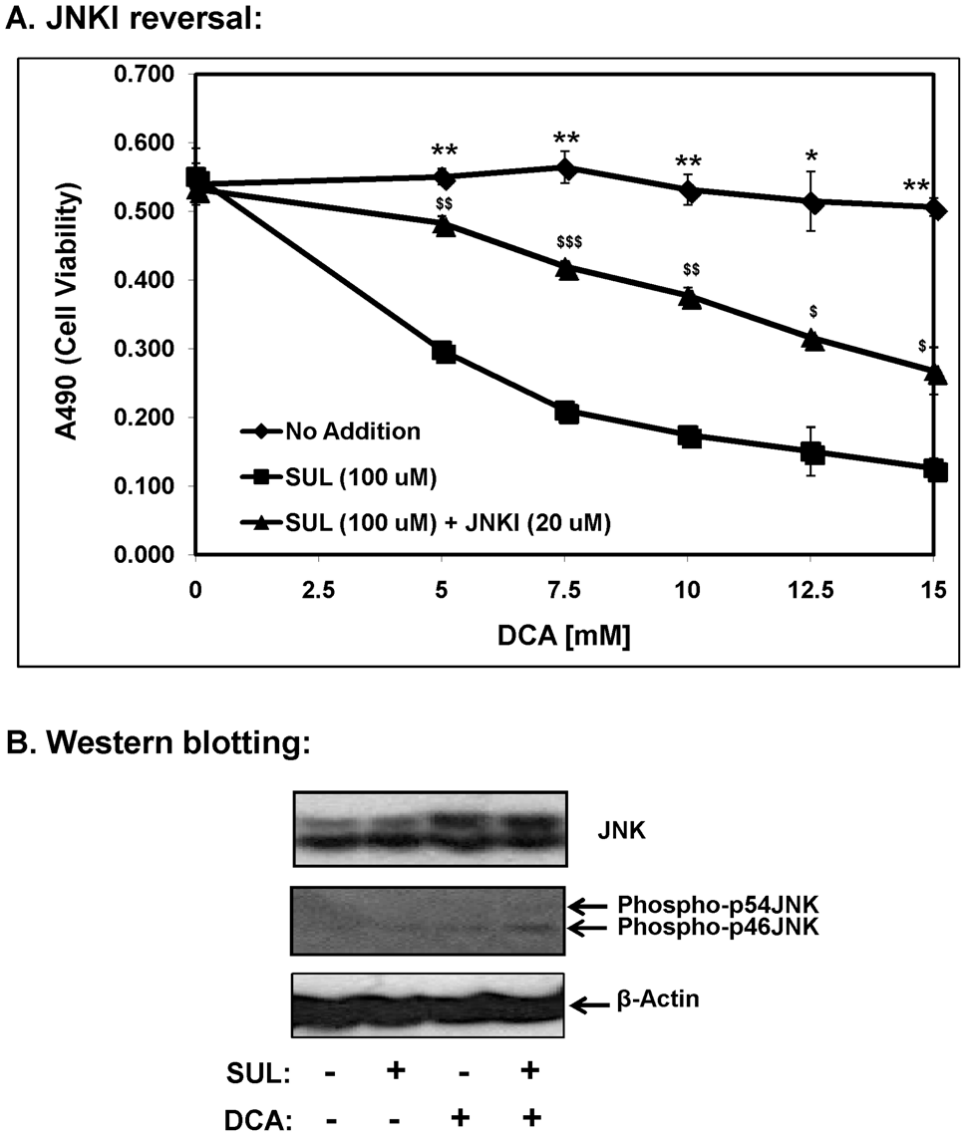
Combination of sulindac and DCA induced apoptosis involves JNK activation. **A**. SCC25 cells were treated for 48 h with sulindac (100 µM) and the indicated concentrations of DCA and where indicated 20 µM of the JNK inhibitor, SP600125. ♦, No drug; ▪, sulindac; ▴, sulindac and SP600125. The cell viability was monitored by MTS assay as mentioned in the Methods. Error bars are standard error of the mean (SEM) expressed as % of the mean value of quadruplicates from a representative experiment. See text for details. Statistical analysis between ♦, No addition and ▪, Sul; *p<0.05, **p<0.005, ***p<0.0005. Statistical analysis between ▪, Sul and ▴, sulindac and JNKI; ^$^p<0.05, ^$$^p<0.005, ^$$$^p<0.0005. **B**. Representative western blots depicting the effect of sulindac and DCA on the levels of total JNK, phopsho-p46JNK and phopsho-p54JNK. β-actin levels were used as an internal control. Cytosolic fractions from SCC25 cells were isolated at the 12 h time point and the levels of JNK and phospho-JNK were determined by western blotting. In the SCC25 cell line, total JNK showed two distinct bands at 46 and 54 kDa.

### Statistical Analysis and Determination of Combination Indices

Data are presented as mean ± SEM for the cell viability assays. For statistical analysis, Minitab statistical software was used to perform Student’s t-test and values with p<0.05 were considered statistically significant. To ascertain the synergistic effect of sulindac and DCA on A549 and SCC25 cancer cell lines, a quantitative analysis of dose-effect relationship was performed to determine the combination indices [26]. Both sulindac and DCA were tested alone on A549 and SCC25 cells at the concentrations indicated. For A549 cells, a ratio of 1∶50 was maintained for the sulindac:DCA drug combinations ranging from 0.2 mM:10 mM up to 1 mM:50 mM, respectively. For SCC25 cells, a ratio of 1∶100 was maintained for the sulindac:DCA drug combinations ranging from 0.05 mM:5 mM up to 0.3 mM:30 mM, respectively. Our experimental results and the determined combination index values are included in the text.

## Results

### Sulindac and DCA Cause Enhanced Killing of A549 and SCC25 Cancer Cells, but not Normal Cells

For these studies we tested the combination of sulindac and DCA on A549 and SCC25 cancer cells. The cells were incubated with each compound alone or in combination for 48 hours before assaying for viability (see Methods). A sulindac dose response curve under these conditions indicated that A549 and SCC25 cancer cells can tolerate a maximum concentration of 500 µM and 100 µM of sulindac, respectively, without exhibiting any significant killing (data not shown), and these concentrations were used in all the studies. DCA, when added, was used at concentrations from 0–40 mM, as indicated. We used these concentrations based on previous reports, which indicated that above 5 mM is required to cause mitochondrial dysfunction in *in vitro* experiments [27]. As shown in Figure 1A, DCA alone (no sulindac) is somewhat toxic to A549 cancer cells, especially above concentrations of 20 mM, but in the presence of sulindac there is enhanced killing of these cells at DCA concentrations above 5 mM. In the case of the SCC25 cancer cells some loss of cell viability with DCA alone was seen even at DCA concentrations below 10 mM (Figure 1B). However, in the presence of sulindac there was again a marked increase in cell death that was clearly evident between DCA concentrations of 2–10 mM. Previously we showed that the combination of sulindac and an oxidizing agent was selective for cancer cells and did not enhance the killing of normal cells [7]. Sulindac and DCA also did not enhance the killing of normal lung and skin cells under the experimental conditions used, as shown in Figures 1C and D. It should be noted that the MRC-5 (lung normal) cells are especially sensitive to DCA, as reported previously [28], for reasons that are not known.

To verify that there was a synergistic effect when the drug combination was used, we determined the combination indices by performing a quantitative analysis of dose-effect relationship [26] on two different cancer cell lines (Figure S1). The combination indices were 0.84 for the A549 and 0.73 for the SCC25 cancer cells, respectively. A value less than 1.00 indicates a synergistic cancer killing effect (Figure S2).

### The Sulindac Effect is not due to its NSAID Activity

In previous studies using sulindac and an oxidizing agent it was shown that the enhanced and selective killing of cancer cells by sulindac and an oxidizing agent was not related to the known NSAID ability of sulindac. To determine the role of COX inhibition a sulindac metabolite, sulindac sulfone, can be used, since it does not inhibit COX 1 or 2 [7], [29]. As shown in Figure 2, using both A549 (A) and SCC25 (B) cancer cells, the combination of sulindac sulfone and DCA showed a similar killing effect as seen above with sulindac. These results indicate that the sulindac enhanced cancer killing effect in the presence of DCA is not related to its known anti-inflammatory activity.

### The Combination of Sulindac and DCA Generate ROS

The synergistic effect on viability observed with sulindac and dichloroacetate with both A549 and SCC25 cancer cells is strikingly similar to previous studies using the combination of sulindac and TBHP [7]. To determine whether ROS production was involved in the selective killing observed in the present studies, the production of ROS, using the indicator dye H_2_DCFDA (see Methods), was determined in the cancer cell lines exposed to sulindac and DCA. The results are summarized in Figure 3. Figure 3A shows the results with A549 cancer cells. It is evident from the results depicted in Figure 3A that untreated A549 cancer cells (panel A1), or cells treated with sulindac alone (panel A2), or DCA alone (panel A3), show only a few positively stained cells. However, when the cells were exposed to both sulindac and DCA (panel A4), a large increase in positively stained cells for ROS (green fluorescence) is seen, showing that the presence of both sulindac and DCA results in the generation of significant levels of ROS. As shown in Figure 3B similar results are seen with the SCC25 cancer cells. Sulindac or DCA alone result in a small increase in ROS producing cells (panels B2 and B3), but a large increase in ROS production is again observed when both drugs are added (panel B4). Quantification using SCC25 cells shows that the number of DCF-positive cells (see Methods) is 9–10× more when the cells are treated with sulindac and DCA as compared to each of the drugs alone (see Figure S3A). It appears from these results and earlier studies that ROS production may be a common feature in the enhanced killing of cancer cells when sulindac is used in combination with compounds that affect mitochondrial function.

### Sulindac in Combination with DCA Results in a Loss of Mitochondrial Membrane Potential

If ROS production is involved in the sulindac/DCA enhanced killing effect one would expect that the production of ROS by the drug combination would affect mitochondrial function. In order to determine this, mitochondrial membrane potential was measured using JC-1 staining as described in Methods. A loss of membrane potential is indicated by an increase in green fluorescence as described in Methods. A typical result is summarized in Figure 4. Both A549 and SCC25 cancer cells were exposed to sulindac and DCA either alone or in combination for 48 hrs and stained with JC-1 in order to monitor the mitochondrial membrane potential. Figure 4A shows the results with the A549 cancer cell line. In the absence of any drug, the mitochondria appear intact and maintain their membrane potential as indicated by little green fluorescence (panel A1). In the presence of sulindac alone (panel A2) or DCA alone (panel A3) there is a small increase in green fluorescence, indicating some loss of mitochondrial membrane potential. However, when both sulindac and DCA are present there is a striking loss of mitochondrial membrane potential as evidenced by a large increase in the green fluorescence (panel A4). We observed the same pattern when several independent fields were analyzed by fluorescent microscopy. Figure 4B shows similar results with the SCC25 cancer cells. Once again a significant loss of mitochondrial membrane potential was only seen when the cells were exposed to both sulindac and DCA (panel B4). Quantification of the effect is shown in Figure S3B. It can be seen that the percent of JC1-green positives cells when the drug combination was used is 3–4× that seen with either drug alone.

### ROS are Involved in the Killing of Cancer Cells by the Combination of Sulindac and DCA

To provide more direct evidence that the ROS produced are involved in the enhanced killing of the cancer cells by sulindac and DCA, we have used two known ROS scavengers, N-acetylcysteine (NAC) and Tiron (see Methods). The results using NAC are shown in Figure 5. Figure 5, panel A, shows that at both 20 and 30 mM DCA, the enhanced killing of A549 cancer cells observed in the presence of sulindac, is largely prevented if NAC (2 mM) is present during the 48 hour incubations. Very similar results are seen with the SCC25 cancer cells as shown in Figure 5, panel B. Comparable results were obtained when Tiron was used in place of NAC (Figure S4).

### Sulindac and DCA Killing of Cancer Cells Involves Apoptotic Death

The results above (Figures 3, 4, 5) show that the enhanced killing of the cancer cell lines involves mitochondrial dysfunction, which suggest that the observed cell death is via apoptosis. Previous studies have indicated that sulindac and its derivatives are proapoptotic drugs [5], [6]. There are also reports that DCA can cause cell death by apoptosis [20], [23]. To determine whether killing of the cancer cells by the combination of these two drugs, mediated by ROS, involves apoptotic death we performed TUNEL staining to measure apoptosis (see Methods). Multiple replicates were tested for sulindac and DCA alone, or in combination, for the TUNEL staining experiments. A typical result is illustrated in Figure 6, where the top panels (Figure 6A, panels A1–A4) represent the results with the A549 cancer cells and the bottom panels (Figure 6B, panels B1–B4) depict the results with the SCC25 cancer cells. When the cells were treated with no drug, sulindac alone, or DCA alone (Figure 6, panels A1–A3 and B1–B3), only a few TUNEL-positive cells are observed. However, when the cells were exposed to both sulindac and DCA, there is a significant increase in TUNEL-positive apoptotic cells (Figure 6, panels A4 and B4), indicating a large induction of apoptosis. To verify the TUNEL results, a more sensitive ligation-mediated PCR-based DNA laddering assay was also used to monitor apoptosis [25]. The results also showed the presence of an enriched strong nucleosomal ladder only when both sulindac and DCA were used in combination (Figure S5; lanes 4 and 8), which strongly supports the TUNEL assay data.

### Sulindac and DCA Killing Involves Proapoptotic JNK Signaling

Of the known mitogen activated protein kinases (MAP kinases), the stress-induced kinase, c-Jun N-terminal kinase (JNK/SAPK) has been directly implicated in apoptotic cell death [11]. Therefore we investigated the role of JNK signaling in sulindac-DCA mediated apoptosis by using SP600125, a JNK-specific inhibitor (JNKI) and these results are presented in Figure 7A. As shown above, SCC25 cells treated with sulindac showed enhanced death in the presence of increasing DCA concentrations. However, when these cells were incubated with sulindac along with SP600125, sulindac-DCA mediated cell death was largely prevented. These results indicate the participation of JNK mediated proapoptotic signaling in the sulindac-DCA mediated cell death.

By western blot analysis, we also determined that the combination of sulindac and DCA significantly increased the levels of phospho-JNK in cytosolic fractions 12 h after the cells were exposed to sulindac and DCA (Figure 7B). An increase in the levels of total JNK (protein bands at 46 and 54 kDa) was seen when the cells were treated with DCA alone as well as when the cells were treated with the combination of sulindac and DCA. It should be noted that both phospho-p46JNK and phospho-p54JNK isoforms were induced by the combination of sulindac and DCA treatment, although the increase in phospho-p46JNK was more significant (Figure 7B).

There is a body of evidence suggesting that JNK initiates release of apoptosis inducing factors from mitochondria, such as cytochrome *c*, that lead to cleavage of caspases and PARP (poly(ADP-ribose) polymerase) [30], [31]. Studies have also shown that during apoptosis, the cytochrome *c* released from mitochondria into the cytoplasm ultimately enters into the nucleus [32]. Our results indicated maximum activation of JNK occurred around 12 h after exposure to sulindac and DCA. This appears to result in the translocation of cytochrome *c* into the cytoplasm and cleavage of PARP 18 h after initial treatment with sulindac and DCA (Figure S6A). As a positive control for these experiments we treated cells with 100 µM of etoposide, an apoptosis-inducing agent. Under sulindac and DCA combination treatment, enhanced nuclear fluorescence can be observed in a majority of cells that are actively undergoing apoptosis (Figure S6B).

Detailed analysis of whole cell immunofluorescence experimental data revealed that ∼94% of cells not treated with either sulindac or DCA showed punctate, mitochondrial cytochrome *c* fluorescence with little diffuse staining in the cytoplasm or in the nuclei. In contrast, after sulindac treatment, 81% of cells showed diffuse, distinct cytoplasmic fluorescence, and very little nuclear fluorescence. After DCA treatment, ∼83% of cells showed diffuse, distinct cytoplasmic fluorescence, and <5% of the cells showed strong nuclear fluorescence. However, when the cells were treated with both sulindac and DCA, ∼72% of cells showed both nuclear and cytoplasmic fluorescence and ∼11% of cells showed strong nuclear fluorescence. These results suggest that the released cytochrome *c* from the mitochondria may initiate the intrinsic apoptotic pathway functioning in the sulindac and DCA mediated cancer killing.

## Discussion

The present study is an extension of our previous work, which demonstrated that sulindac made cancer cells, but not normal cells, more sensitive to oxidative stress [7]. In these previous experiments sulindac was pre-incubated with the cells for 24–48 hours and then the sulindac was removed before the cells were exposed to either TBHP or H_2_O_2_ for 2 hours. It was evident from the previous experiments that sulindac pretreatment made the cancer cells much more sensitive to the oxidizing agent resulting in a large increase in ROS and loss of mitochondrial function [7].

It seemed reasonable, based on these results, that sulindac in combination with compounds that affected mitochondrial respiration would result in selective enhanced killing of cancer cells, but not normal cells. In the present study using A549 and SCC25 cancer cell lines, the combination of sulindac and DCA enhanced the killing of these cancer cell lines, but not normal lung or skin cells. Our results on the amounts of DCA needed in whole cells are consistent with what has been reported previously [28], [33], [34]. In our system, the IC50 for DCA with SCC25 cells is 23 mM and for A549 cells is 35 mM. The IC50 for normal keratinocytes is >50 mM and for normal lung cells (MRC5) is ∼40 mM. The results also indicated that the cancer cell death that was observed involves ROS production, JNK activation, and mitochondria initiated apoptosis. With regard to a lack of effect on normal cells, it has been shown that sulindac protects normal lung cells against oxidative damage resulting from TBHP exposure [7] and we have also recently reported sulindac can protect cardiac cells against oxidative damage resulting from ischemia/reperfusion through a preconditioning mechanism [35].

To our knowledge there are now at least 8 compounds, including our studies with TBHP, H_2_O_2_ and DCA, that have shown enhanced and selective cancer killing in the presence of sulindac [7]–[9], [12]–[15]. Although their metabolic targets within the cell are known, and are different, it is quite likely that they all, directly or indirectly, cause cell death in the presence of sulindac through a mechanism that involves an alteration in mitochondrial respiration and ROS production [10], [12], [14]. It seems likely that when one finds a drug, that in combination with sulindac, selectively kills cancer cells, but not normal cells, the mechanism of killing involves oxidative stress leading to mitochondrial dysfunction. Altered respiration may be a common factor in these experiments using sulindac/drug combinations and the present results using DCA support this view. It is quite possible that the sulindac effect may be related to the observations made more than 50 years ago by Warburg, who noted that normal cells prefer respiration to obtain their energy, whereas cancer cells prefer glycolysis, due to a defect in the respiratory chain [16]. This basic difference in mitochondrial respiration between normal and cancer cells may make cancer cells more sensitive to oxidative stress [36], [37]. It seems that sulindac may amplify this fundamental difference in the biochemistry of normal and cancer cells. Although how sulindac sensitizes cancer cells to drugs that affect mitochondrial respiration is still not clear, but is under active investigation. Spitz and coworkers [38], in studies on glucose deprivation of cancer cells, have come to a similar conclusion regarding the differences in metabolism between normal and cancer cells. In line with these results, another recent study has shown that pharmacological inhibition of lactate dehydrogenase could result in selective cancer killing [39]. In the latter studies it was shown that the enhanced selective killing of cancer cells also involved ROS production, and the effect seen was attributed to an altered respiratory process in the cancer cells.

It should be pointed out that the combination of sulindac with an oxidizing agent or drugs that may affect mitochondrial function has already been tested clinically. Meyskens et al. 2008 showed that the combination of sulindac with DFMO had a significant effect on the recurrence of colon polyps and appearance of colon cancer in a 3-year clinical study [13]. We recently reported the use of sulindac, with H_2_O_2_, in a proof of concept clinical study for the topical treatment of actinic keratoses [15]. One of the disadvantages of using this combination was the need for two topical formulations since the compounds could not be stored for long periods without destruction of the sulindac by the H_2_O_2_. In addition, one cannot use H_2_O_2_ for treatment of internal tumors since it cannot be taken orally. However, the combination of sulindac and DCA could be delivered as a single formulation amenable for topical use, and the two compounds can be used orally. In fact, for several years, DCA has been used clinically to lower lactic acid levels in patients suffering from lactic acidosis [40]–[42]. DCA also has been used as an anti-cancer agent *in vitro* and *in vivo* using several different cancer cell lines indicating that mitochondrial metabolism in cancer cells could be a new therapeutic target [18], [20], [22]. Michelakis et al., (2010) have shown that treatment with DCA “remodels” mitochondrial metabolism in glioblastoma patients with reversible toxic effects. It should be noted that both sulindac and DCA are affordable, relatively non-toxic and can be taken orally. If the combination proves to be successful *in vivo* it will add a new dimension in cancer treatment as both the drugs target mitochondrial metabolism in multiple cancers [22].

In summary, our studies using the combination of sulindac and DCA suggest that sulindac selectively makes cancer cells more sensitive to agents that affect mitochondrial respiration resulting in oxidative stress and mitochondrial dysfunction. These results could be related to the respiration defect in cancer cells, originally observed by Warburg [16]. Studies aimed at understanding the fundamental differences between how cancer cells and normal cells respond to sulindac and agents that affect mitochondrial function are currently under investigation.

## Supporting Information

Figure S1 **Cytotoxicity of sulindac, DCA, or drug combination on A549 and SCC25 cancer cells.** The A549 and SCC25 cancer cells were treated with the indicated concentrations of sulindac (panels 1 and 4) or DCA (panels 2 and 5). In the sulindac/DCA drug combination experiments, a ratio of 1∶50 (sulindac:DCA) was maintained for the A549 cells, with drug combinations ranging from 0.2 mM:10 mM up to 1 mM:50 mM (panel 3) and a ratio of 1∶100 (sulindac:DCA) was maintained for the SCC25 cells with drug combinations ranging from 0.05 mM:5 mM up to 0.3 mM:30 mM (panel 6).
(TIF)

Figure S2 **Determination of combination indices for A549 and SCC25 cancer cells.** The drug combination indices were determined by incorporating the cell viability values obtained above into the equations of Chou and Talalay [26]. See text for further details.
(TIF)

Figure S3 **Quantification of DCF and JC-1 positive green fluorescent cells.** SCC25 cells were treated with sulindac, DCA, or drug combination for 48 hrs and stained with H_2_DCFDA (Fig. S3A) or JC-1 (Fig. S3B) dyes as mentioned in Methods. For positive control, the cells were treated with 200 µM of TBHP for 2 hrs and staining performed as above. The cells were analyzed under high power magnification using 100× objective in an Olympus inverted fluorescent microscope. At least 100 individual cells were visualized for each condition and the percentages of DCF-positive and JC1-positive green fluorescent cells are presented in tabular and graphical formats.
(TIF)

Figure S4 **The ROS scavenger Tiron reverses the killing of cancer cells by the combination of sulindac and DCA.** The A549 and SCC25 cancer cells were treated with the indicated concentrations of DCA in the absence (grey bar) or presence of sulindac (black bar) or presence of sulindac and Tiron (striped bar) for 48 hours. The cell viability was monitored by MTS assay as mentioned in the Methods. The cell viability is expressed as % of control (cells not treated with sulindac). Error bars are standard error of the mean (SEM) expressed as % of the mean value of quadruplicates from a representative experiment. Inhibition of cancer cell growth occurred in a dose dependent manner during combination treatment of DCA and sulindac (black bars) in both A549 cancer (A) and SCC25 cancer cells (B). However, this enhanced killing was prevented when Tiron was present along with the drug combination treatment (striped bars in A and B).
(TIF)

Figure S5 **Pronounced nucleosomal DNA laddering occurs during the killing of cancer cells by the combination of sulindac and DCA.** The A549 and SCC25 cancer cells were treated with the indicated drugs for 48 hours. Nucleosomal DNA was extracted and subjected to ligation-mediated PCR as described in Methods and analyzed on a 1.2% agarose gel along with size markers. Lane ‘M’ denotes molecular size markers. Lanes 1–4 and 5–8 depict the results obtained with A549 cancer and SCC25 cancer cells respectively. Results are illustrated in lanes 1 and 5 (no drug), lanes 2 and 6 (sulindac alone), lanes 3 and 7 (DCA alone), and lanes 4 and 8 (sulindac and DCA). An enhanced nucleosomal DNA laddering was observed only with sulindac and DCA drug combination treatment (lanes 4 and 8).
(TIF)

Figure S6 **Combination of sulindac and DCA leads to release of cytochrome** ***c*** **from mitochondria and cleavage of PARP.** SCC25 cells were treated with sulindac, DCA, drug combination, or etoposide to assay for the intra-cellular location of cytochrome *c* by western blotting and immunofluorescence. **A.** Cytosolic fractions were isolated at 18 h and the presence of cytochrome *c* in the cytoplasm and cleavage of PARP was determined by western blotting. Representative western blots show the amount of cytochrome *c* and cleaved PARP. β-actin levels were used as an internal control. **B.** Immunofluorescence was performed using the CBA077 InnoCyte™ Flow Cytometric Cytochrome *c* Release Kit according to the manufacturer’s instructions. Several independent fields were analyzed and the representative micrographs show the localization patterns of cytochrome *c* when the cells are exposed to sulindac and/or DCA. Quantitative values are presented in the text.
(TIF)
