# Supplementary figures and images for: Combination of Sulindac and Dichloroacetate Kills Cancer Cells via Oxidative Damage
Source: PLoS One. 2012 Jul 17;7(7):e39949. doi: 10.1371/journal.pone.0039949 (PMC3398923; doi:10.1371/journal.pone.0039949)

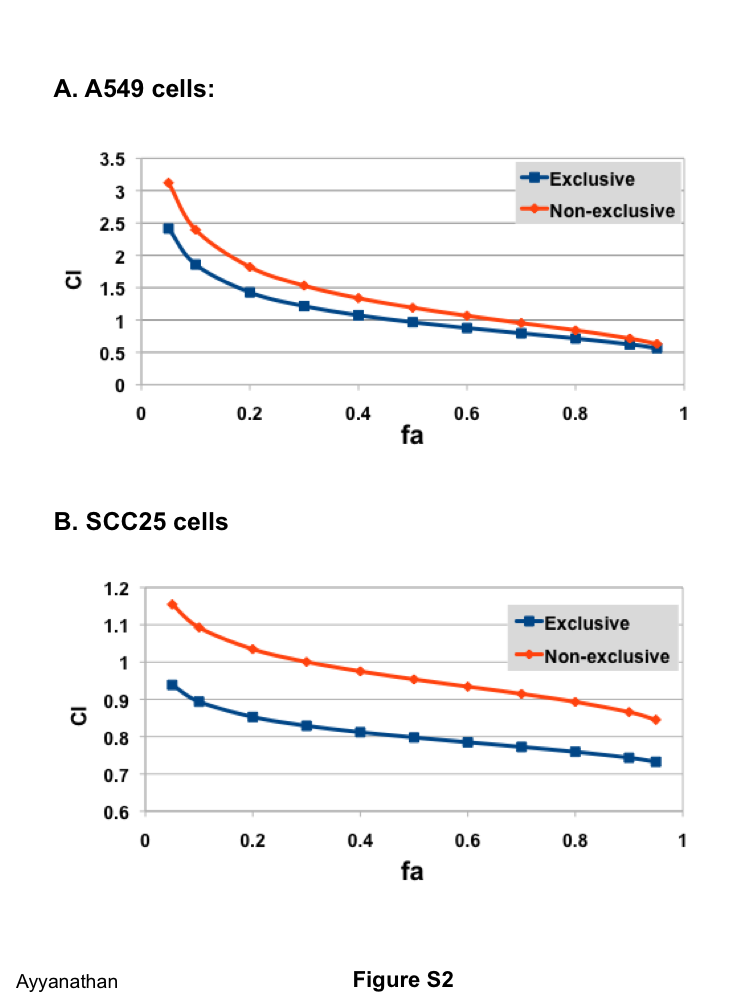

Supplement: Figure S2 — Determination of combination indices for A549 and SCC25 cancer cells. The drug combination indices were determined by incorporating the cell viability values obtained above into the equations of Chou and Talalay [26]. See text for further details. (TIF) [file pone.0039949.s002.tif]

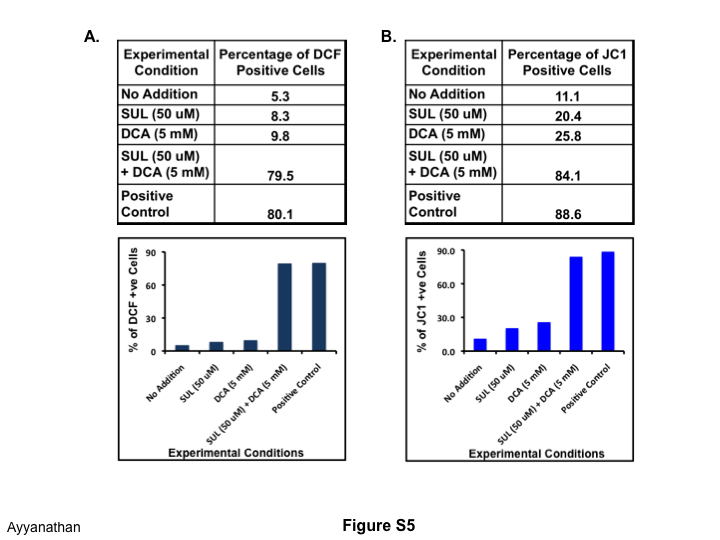

Supplement: Figure S3 — Quantification of DCF and JC-1 positive green fluorescent cells. SCC25 cells were treated with sulindac, DCA, or drug combination for 48 hrs and stained with H2DCFDA (Fig. S3A) or JC-1 (Fig. S3B) dyes as mentioned in Methods. For positive control, the cells were treated with 200 µM of TBHP for 2 hrs and staining performed as above. The cells were analyzed under high power magnification using 100× objective in an Olympus inverted fluorescent microscope. At least 100 individual cells were visualized for each condition and the percentages of DCF-positive and JC1-positive green fluorescent cells are presented in tabular and graphical formats. (TIF) [file pone.0039949.s003.tif]

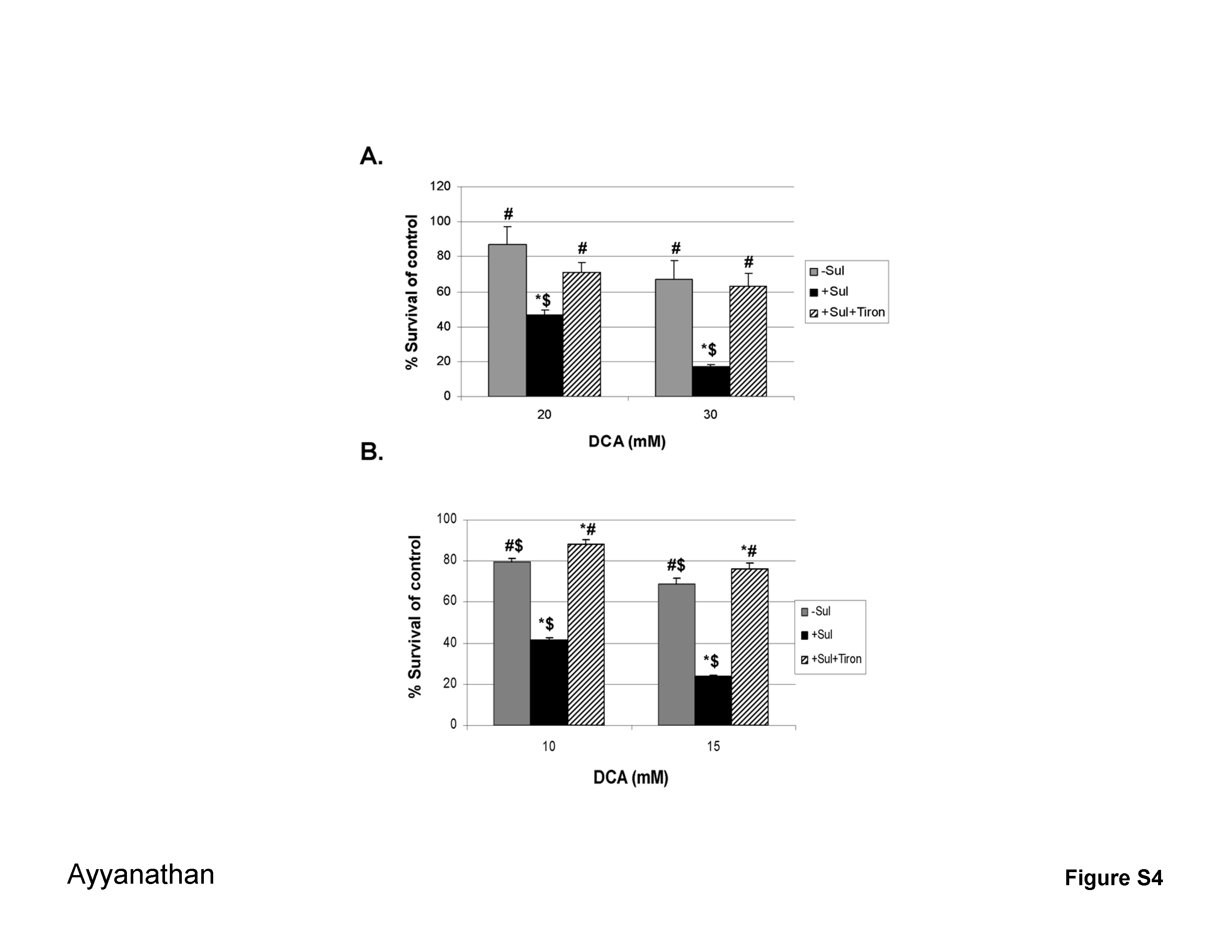

Supplement: Figure S4 — The ROS scavenger Tiron reverses the killing of cancer cells by the combination of sulindac and DCA. The A549 and SCC25 cancer cells were treated with the indicated concentrations of DCA in the absence (grey bar) or presence of sulindac (black bar) or presence of sulindac and Tiron (striped bar) for 48 hours. The cell viability was monitored by MTS assay as mentioned in the Methods. The cell viability is expressed as % of control (cells not treated with sulindac). Error bars are standard error of the mean (SEM) expressed as % of the mean value of quadruplicates from a representative experiment. Inhibition of cancer cell growth occurred in a dose dependent manner during combination treatment of DCA and sulindac (black bars) in both A549 cancer (A) and SCC25 cancer cells (B). However, this enhanced killing was prevented when Tiron was present along with the drug combination treatment (striped bars in A and B). (TIF) [file pone.0039949.s004.tif]

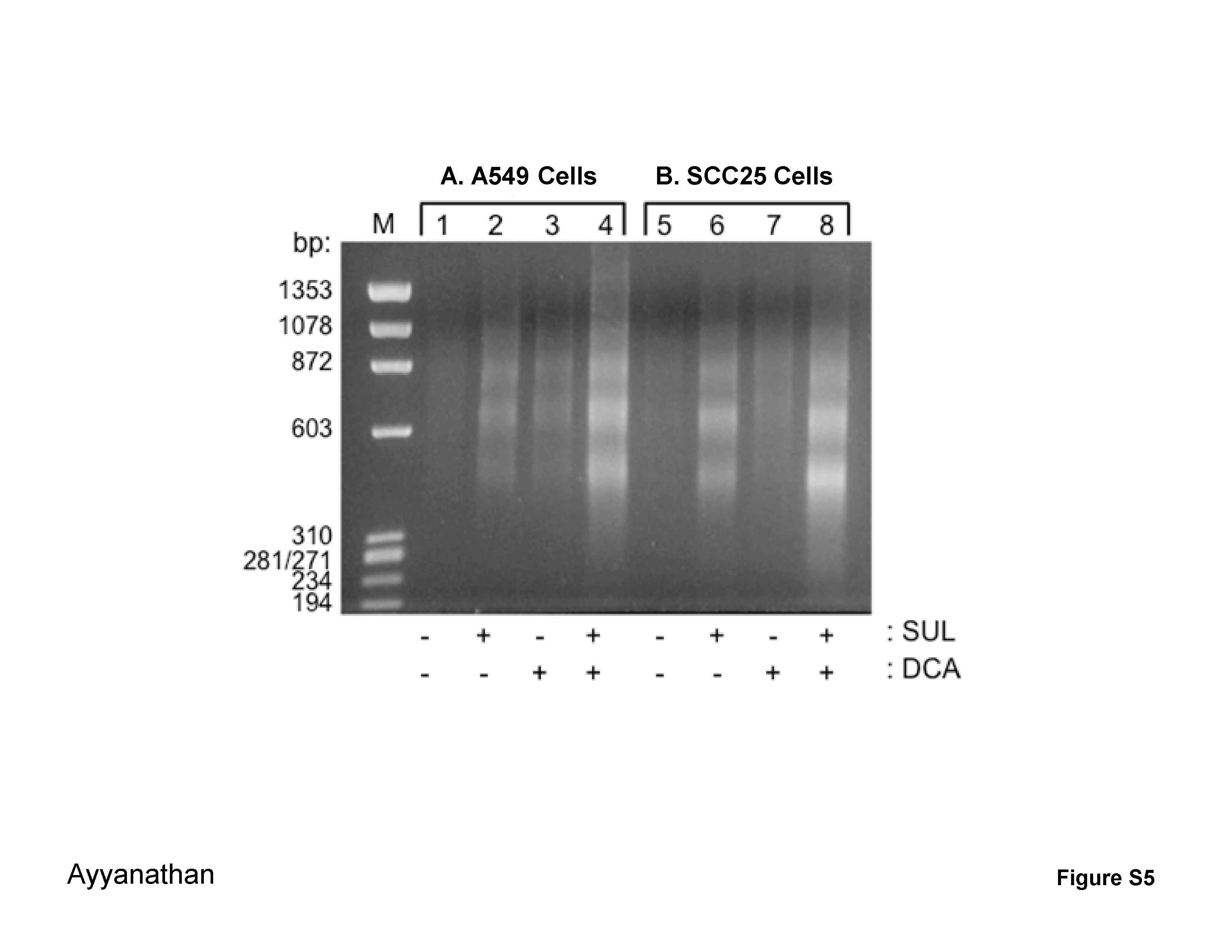

Supplement: Figure S5 — Pronounced nucleosomal DNA laddering occurs during the killing of cancer cells by the combination of sulindac and DCA. The A549 and SCC25 cancer cells were treated with the indicated drugs for 48 hours. Nucleosomal DNA was extracted and subjected to ligation-mediated PCR as described in Methods and analyzed on a 1.2% agarose gel along with size markers. Lane ‘M’ denotes molecular size markers. Lanes 1–4 and 5–8 depict the results obtained with A549 cancer and SCC25 cancer cells respectively. Results are illustrated in lanes 1 and 5 (no drug), lanes 2 and 6 (sulindac alone), lanes 3 and 7 (DCA alone), and lanes 4 and 8 (sulindac and DCA). An enhanced nucleosomal DNA laddering was observed only with sulindac and DCA drug combination treatment (lanes 4 and 8). (TIF) [file pone.0039949.s005.tif]

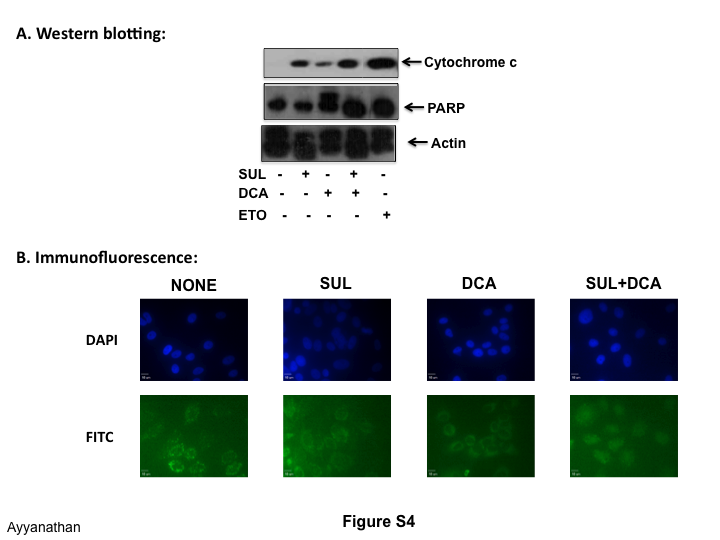

Supplement: Figure S6 — Combination of sulindac and DCA leads to release of cytochrome c from mitochondria and cleavage of PARP. SCC25 cells were treated with sulindac, DCA, drug combination, or etoposide to assay for the intra-cellular location of cytochrome c by western blotting and immunofluorescence. A. Cytosolic fractions were isolated at 18 h and the presence of cytochrome c in the cytoplasm and cleavage of PARP was determined by western blotting. Representative western blots show the amount of cytochrome c and cleaved PARP. β-actin levels were used as an internal control. B. Immunofluorescence was performed using the CBA077 InnoCyte™ Flow Cytometric Cytochrome c Release Kit according to the manufacturer’s instructions. Several independent fields were analyzed and the representative micrographs show the localization patterns of cytochrome c when the cells are exposed to sulindac and/or DCA. Quantitative values are presented in the text. (TIF) [file pone.0039949.s006.tif]
